# Crosswise Stream of Cu-H_2_O Nanofluid with Micro Rotation Effects: Heat Transfer Analysis

**DOI:** 10.3390/nano13030471

**Published:** 2023-01-24

**Authors:** Rashid Mehmood, Rabil Tabassum, Mohamed R. Ali, Taseer Muhammad

**Affiliations:** 1 Department of Mathematics, Faculty of Natural Sciences, HITEC University, Taxila Cantt, Taxila 47080, Pakistan; 2 Department of Mathematics, Faculty of Basic and Applied Sciences, Air University, Islamabad 44000, Pakistan; 3 Center of Research, Faculty of Engineering and Technology, Future University in Egypt, New Cairo 11835, Egypt; 4 Department of Mathematics, College of Science, King Khalid University, Abha 61413, Saudi Arabia

**Keywords:** micro-rotation, copper nanoparticles, oblique flow, numerical scheme

## Abstract

The present study focuses on a crosswise stream of liquid-holding nano-sized particles over an elongating (stretching) surface. Tiny particles of copper are added into base liquid (water). The influence of the micro rotation phenomenon is also considered. By means of appropriate transformations non-linear coupled ordinary differential equations are attained that govern the flow problem. The Runge–Kutta–Fehlberg scheme, together with the shooting method, is engaged to acquire results numerically. Micropolar coupling parameter, microelements concentration and nanoparticles volume fraction effects are examined over the profiles of velocity, temperature and micro-rotation. Moreover, heat flux and shear stress are computed against pertinent parameters and presented through bar graphs. Outcomes revealed that material constant has increasing effects on normal components of flow velocity; however, it decreasingly influences the tangential velocity, micro-rotation components and temperature profile. Temperature profile appeared to be higher for weak concentration of microelements. It is further noticed that normal velocity profile is higher in magnitude for the case of strong concentration (n = 0) of microelements, whereas tangential velocity profile is higher near the surface for the case of weak concentration (n = 0.5) of microelements. An increase of 3.74% in heat flux is observed when the volume fraction of nanoparticles is increased from 1 to 5%.

## 1. Introduction

Heat transferal properties of fluids are highly influenced by their thermal conductivity. Metals have higher thermal conductivity than liquids. This property of metals inspired Choi et al. [1] to suggest the insertion of metallic nanoparticles in traditional fluids to improve their rate of heat transfer. Over the past decade or so, nanofluids have become an essential feature in almost all modern day industrial and technological devices and procedures. Nano particles are easily suspended within base fluids to keep the mixture homogenous while making it a lot more effective when it comes to their thermal capabilities. Several manufacturing procedures operating at high temperatures are required to dissipate heat at a reasonably rapid pace. Nanofluids can be a promising solution to all these aspects and challenges. Keeping this interesting property in view, many researchers pondered over the flow of nanoliquid. A flow phenomenon in which fluid strikes a solid wall or surface is called stagnated flow. This type of flow can be orthogonal or transverse. For the first time Hiemenz [2] reported the flow problem towards point of stagnation. Mustafa et al. [3] reported flow characteristics of nanoliquid in the neighborhood of the stagnated point over an extending sheet. Solutions are developed by homotopic analysis. A rise in Nusselt number is observed with stretching ratio. Hamad et al. [4] performed an analysis via the Lie group for the flow of the boundary layer stagnation flow problems above a widening permeable sheet. They examined the influential characteristics of generation and absorption of heat over the flow of a nanoliquid that saturates the permeable sheet. Ibrahim et al. [5] made an analysis on flow towards point of stagnation with magnetic field upon an elongating sheet. They suggested that surface heat transfer rises when magnetic parameter grows. It is also noted that velocity ratio parameter caused a rise in skin friction. Malvandi et al. [6] implemented no-slip condition at a boundary to examine its impact on the time-dependent flow of a nanoliquid containing various types of nanoparticles towards the point of stagnation upon an extending surface. It was discovered that the rate of heat transfer is intensified by slip and stretching parameters. An analysis of time-independent Casson model based non-Newtonian fluid with the inclusion of nano-sized particles near a point of stagnation flowing towards an enlarging surface was presented by Nadeem et al. [7]. The formation of boundary layer is found for the situation when in-viscous free stream velocity is higher compared with stretching sheet velocity of the Casson fluid constant, which caused a decline in the velocity of fluid at a particular point. Sheikholeslami et al. [8] inspected the flow features of nanoliquid flowing between two plates placed parallel horizontally in a system with rotation. Their assessment showed that skin friction was increasingly influenced by Reynold number, as well as rotation constant, and they also perceived that heat flux decayed with Eckert number. Nadeem et al. [9] proposed the non-aligned flow of the Walter-B form of nanoliquid passing above a surface with convective condition and transversely applied magnetic field. They revealed that components of shear stress are oppositely influenced by magnetic field. Moreover, mass and heat transfusion rates decayed with magnetic field strength. Javed et al. [10] investigated a time-dependent stagnated flow transversely striking an oscillatory levelled plate with magnetic field applied uniformly. Finite difference method, implicit in nature, was used for solving the problem. Translation in the transverse stagnated point was noted with magnetic field. Amir [11] studied the heat transferal properties of nanofluids above a sphere with rotation dependent upon time. A continuous variation in the velocity of free stream is considered with respect to time. It was revealed that concentration and heat transfer rates fall down with thermophoresis constant. Nadeem et al. [12] considered the fluid containing carbon nano-tubes to be transversely striking a point of stagnation over a convective extending sheet. Results proved that engine-oil-based nanoliquid showed a comparatively better rate of heat transfer than a water-based nanoliquid. Rehman et al. [13] deliberated the stagnation flow of obliquely striking nanofluid to a shrinking surface with non-compressible and viscous properties. An increase in flow obliquity near the surface is discovered for intensified strain rate. Khan et al. [14] testified the effect of variation in viscosity and thermal radiation on transverse stagnation flow of nanoliquid past above the elongating surface, which was heated convectively. A decline in the non-alignment of the re-attachment point is discovered by strengthening the magnetic field. Tabassum et al. [15] pondered over the oblique stagnated flow of water-based nanoliquids with copper nanoparticles and discovered the influence of viscosity, varying with the temperature. Their effort revealed that viscosity parameter positively influenced the rate of heat transfer but reverse impact was found on skin friction.

The micro polar liquid theory, initiated by Eringen [16], is able to describe the behavior of fluids that are very complex in nature, such as lubricants, blood of animals, liquid crystals, shear flows with turbulence, etc. Peddieson [17] did an analysis of microploar liquid flowing on the way to a stagnation point above a flat surface. He proposed a new boundary condition in his work motivated by turbulence theories. Nazar et al. [18] inspected the flow of the stagnation micro polar type of liquid on a sheet extending in its own plane. They used the Keller box numerical scheme to get the required results. Pop et al. [19] investigated the micro polar liquid flow above a penetrable shrinking surface with the influence of thermal radiation. They revealed in their analysis that mass suction at wall increased for the case of steady flow. Sheikholeslami et al. [20] analytically explored the micropolar category of fluid flow within a channel, along with chemical reaction using homotopy perturbation technique. The briefly explained the concept of the technique used for finding the solution of leading equations. Aurangzeb et al. [21] analyzed the significance of a boundary layer micropolar liquid flow over a penetrable exponentially shrinking surface. They obtained a dual type of similarity solutions of micro rotation, velocity and temperature distribution for the case of mass suction. Rashid et al. [22,23,24] have performed comprehensive studies on micro polar liquid flow over an extending surface with changes in viscosity w.r.t viscosity. The influence of deviation in viscosity on the non-aligned stagnated flow of micro polar nanoliquid was studied by Mehmood et al. [22]. They assessed an augmentation in a normal micro-rotation component and temperature profile with growth in variable viscosity parameter, while tangential micro-rotation component and transfer of heat decayed with this parameter.

Hsiao [25] investigated the problem of the stagnation nano energy conversion for conjugate mixed convection heat and mass transfer with electrical magneto hydrodynamic (EMHD) and heat source/sink effects on nanofluid flow field over a slip boundary stretching sheet surface. In another study, Hasio [26] investigated the problem composed of activation energy electrical MHD Ohmic dissipation and the mixed convection of a viscoelastic non-Newtonian Carreau nanofluid on a stagnation point energy conversion. Hsiao also [27] presented a study of numerical application to a thermal extrusion manufacturing processing system energy conversion problem by using an improved parameters control method. In another investigation, Hsiao [28] studied an applied thermal system for heat and mass transfer and the energy management problem of hydromagnetic flow with magnetic and viscous dissipation affecting micropolar nanofluids towards a stretching sheet. Hejri et al. [29] conducted a study for analysis of the natural convection and entropy production in a two-dimensional section of the heat exchanger. The lattice Boltzmann method, which is equipped with a Bhavnagar–Gross–Krook model, is used for this purpose. Rashid et al. [30] introduced a review of experimental, numerical and analytical studies related to heat transfer analyses in different geometries, such as circular, cylindrical, hexagonal and rectangular cavities. Fuxi et al. [31] studied the effects of hybrid nanofluids and helical coil pitch in a 3D shell and tube heat exchanger. They used phase Coupled SIMPLE algorithm. Kamali et al. [32] employed a modern numerical approach for conducting the simulations, which uses the smoothed-profile lattice Boltzmann method. They used two separate distribution functions for flow and temperature fields to solve the Navier–Stokes equations in the most efficient manner. Malekshah et al. [33] analyzed the effect of the absorber’s geometry and operating fluid on the thermal and hydrodynamic behaviors of a solar collector. They proposed two different profiles for the absorber, which are wavy and flat. Abed et al. [34] carried out a comprehensive experimental/numerical investigation for the purpose of heat transfer and hydrodynamic analysis of natural convection within a cavity equipped with active rectangular fins. Numbers of hot and cold fins are fitted at the surface of square cavity, which have remarkable effect on the hydrothermal performance.

Micro-polar fluids are fluids, which couple the macroscopic velocity field and the particles rotational motion. These fluids are made of hard particles, which are suspended in a viscous medium. Ferro-fluids, bubbly liquids and blood flows are some examples of micro-polar fluids. Micro-polar fluids are also used in some industrial applications, such as polymer solutions, lubricant fluids and biological structures. Industrial applications involving potential features of micro polar liquids is our motivation behind this study. Our intention is to solve the transverse flow problem of the micro polar nanofluid near a stagnated point with the insertion of copper nanoparticles. Our focus is to examine the various flow characteristics with influence of micro polar coupling parameter, volume fraction of nanoparticles and concentration of microelements. The flow governing problem is initially presented by utilizing traditional Navier–Stokes theory along with law of the conservation of energy. The prevailing governing equations are transformed using scaling analysis, which afterwards are solved numerically. The physical analysis of the obtained graphical results is presented in due detail. Finally concluding remarks are presented at the end.

## 2. Problem Formulation

**Figure 1:**
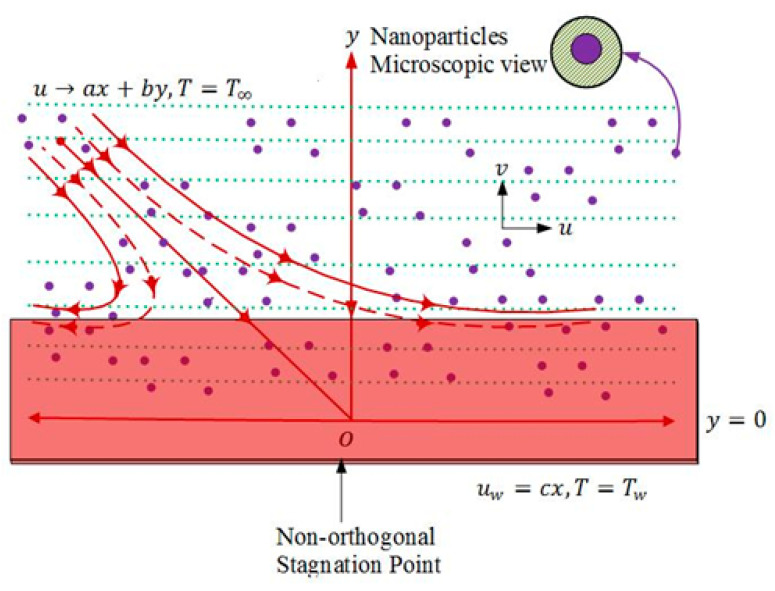
Physical depiction of flow problem.

We are focusing on time-independent, transverse stagnated flow of incompressible micropolar nanoliquid in two dimensions. Two forces were implemented along x-axis with same strength but reverse in direction. These forces were applied with objective of keeping the origin fixed when the surface was stretched, as presented in Figure 1. Fluid is occupying the region above x-axis. Model equations describing the above-mentioned steady flow characteristics with no viscous dissipation are shown as:

(1) $$\frac{\partial {\hat{u}}^{*}}{\partial {\hat{x}}^{*}}+\frac{\partial {\hat{v}}^{*}}{\partial {\hat{y}}^{*}}=0,$$

(2) $${\hat{\rho }}_{nf}{\hat{u}}^{*}\frac{\partial {\hat{u}}^{*}}{\partial {\hat{x}}^{*}}+{\hat{\rho }}_{nf}{\hat{v}}^{*}\frac{\partial {\hat{u}}^{*}}{\partial {\hat{y}}^{*}}+\frac{\partial {\hat{p}}^{*}}{\partial {\hat{x}}^{*}}=\left({\hat{\mu }}_{nf}+\hat{k}\right)\frac{\partial^{2}{\hat{u}}^{*}}{\partial {\hat{y}}^{{*}^{2}}}+\hat{k}\frac{\partial {\hat{N}}^{*}}{\partial {\hat{y}}^{*}},$$

(3) $${\hat{\rho }}_{nf}{\hat{u}}^{*}\frac{\partial {\hat{v}}^{*}}{\partial {\hat{x}}^{*}}+{\hat{\rho }}_{nf}{\hat{v}}^{*}\frac{\partial {\hat{v}}^{*}}{\partial {\hat{y}}^{*}}+\frac{\partial {\hat{p}}^{*}}{\partial {\hat{y}}^{*}}=\left({\hat{\mu }}_{nf}+\hat{k}\right)\frac{\partial^{2}{\hat{v}}^{*}}{\partial {\hat{y}}^{{*}^{2}}}-\hat{k}\frac{\partial {\hat{N}}^{*}}{\partial {\hat{x}}^{*}},$$

(4) $$({\hat{\rho }}_{nf}\cdot j){\hat{u}}^{*}\frac{\partial {\hat{N}}^{*}}{\partial {\hat{x}}^{*}}+({\hat{\rho }}_{nf}\cdot j){\hat{v}}^{*}\frac{\partial {\hat{N}}^{*}}{\partial {\hat{y}}^{*}}=-\hat{k}\left(2{\hat{N}}^{*}+\frac{\partial {\hat{u}}^{*}}{\partial {\hat{y}}^{*}}\right)+{\hat{\gamma }}_{nf}\frac{\partial^{2}{\hat{N}}^{*}}{\partial {\hat{y}}^{{*}^{2}}},$$

(5) $${\hat{u}}^{*}\frac{\partial {\hat{T}}^{*}}{\partial {\hat{x}}^{*}}+{\hat{v}}^{*}\frac{\partial {\hat{T}}^{*}}{\partial {\hat{y}}^{*}}=({\hat{\alpha }}_{nf}^{*})\frac{\partial^{2}{\hat{T}}^{*}}{\partial {\hat{y}}^{{*}^{2}}}$$

Above equations are describing ${\hat{u}}^{*},{\hat{v}}^{*}$ as velocity components in ${\hat{x}}^{*},$ ${\hat{y}}^{*}$ directions, ${\hat{p}}^{*}$ as pressure, thermal diffusivity by ${\hat{\alpha }}_{nf}^{*}=\frac{{\hat{k}}_{nf}}{{\left(\hat{\rho }c_{\hat{p}}\right)}_{nf}}$ and density of nanoliquid as ${\hat{\rho }}_{nf}$. Component of micro rotation vector in normal direction to ${\hat{x}}^{*}-{\hat{y}}^{*}$ plane is symbolized by ${\hat{N}}^{*}$. $j=\frac{{\hat{\nu }}_{f}}{c}$,${\hat{\gamma }}_{nf}=\left({\hat{\mu }}_{nf}+\frac{\hat{k}}{2}\right)j,$ ${\hat{\mu }}_{nf},$ $\hat{k},$ ϕ and ${\hat{T}}^{*}$ denote the micro inertia density, spin gradient viscosity, dynamic viscosity, vortex viscosity, copper nanoparticles volume fraction and temperature of nanofluid. ${\hat{\rho }}_{nf}$ and ${\left(\hat{\rho }c_{\hat{p}}\right)}_{nf}$ are well-defined [22], as below:

(6) $${\hat{\rho }}_{nf}={\hat{\rho }}_{f}+\phi \left({\hat{\rho }}_{s}-{\hat{\rho }}_{f}\right)$$

(7) $${\left(\hat{\rho }c_{\hat{p}}\right)}_{nf}={\left(\hat{\rho }c_{\hat{p}}\right)}_{f}+\phi \left({\left(\hat{\rho }c_{\hat{p}}\right)}_{s}-{\left(\hat{\rho }c_{\hat{p}}\right)}_{f}\right),$$

where, ${\hat{\rho }}_{f}$ and ${\hat{\rho }}_{s}$ denote the density of carried liquid (base liquid) and mass density of copper nanoparticles, respectively. A Maxwell–Garnett model [22] is employed to approximate the ${\hat{k}}_{nf}$, as below:

(8) $${\hat{k}}_{nf}={\hat{k}}_{f}\left(\frac{{\hat{k}}_{s}+2{\hat{k}}_{f}-2\phi {\hat{k}}_{f}+2\phi {\hat{k}}_{f}}{{\hat{k}}_{s}+2{\hat{k}}_{f}+\phi {\hat{k}}_{f}-\phi {\hat{k}}_{s}}\right),$$

where ${\hat{k}}_{s}$ and ${\hat{k}}_{f}$ symbolize the thermal conductivities of copper nanoparticles and water as carrier liquid, respectively. Some properties defining the thermal and physical characteristics of copper and water are specified in Table 1. Conditions at boundaries are:

(9) $${\hat{u}}^{*}=c{\hat{x}}^{*},{\hat{v}}^{*}=0,{\hat{T}}^{*}={\hat{T}}_{w},{\hat{N}}^{*}=-n\frac{\partial {\hat{u}}^{*}}{\partial {\hat{y}}^{*}}\;at\;{\hat{y}}^{*}=0,$$

(10) $${\hat{u}}^{*}=a{\hat{x}}^{*}+b{\hat{y}}^{*},{\hat{T}}^{*}={\hat{T}}_{\infty },{\hat{N}}^{*}=\mathrm{constant},\;\;\;\mathrm{when}\;{\hat{y}}^{*}\to \infty .$$

where constant values are a, b and c, ${\hat{T}}_{w}$ and ${\hat{T}}_{\infty }$ represent the wall temperature and ambient temperature of nanoliquid. It is essential to know that microelements concentration is characterized by n and $n\in \left[0,1\right]$ [22]. Micro-elements concentration appears to be strong if n is taken 0 and for n=0.5 it becomes weak and n = 1 is employed for modelling of turbulence in boundary layer flows, which is not the case considered in the present study. Here, we present the subsequent transformations:

(11) $$\hat{x}=\frac{{\hat{x}}^{*}}{\sqrt{\frac{{\hat{\nu }}_{f}}{c}}},\;\hat{y}=\frac{{\hat{y}}^{*}}{\sqrt{\frac{{\hat{\nu }}_{f}}{c}}},\;\hat{u}=\frac{{\hat{u}}^{*}}{\sqrt{{\hat{\nu }}_{f}c}},\;\hat{v}=\frac{{\hat{v}}^{*}}{\sqrt{{\hat{\nu }}_{f}c}},\;\hat{p}=\frac{{\hat{p}}^{*}}{{\left(\hat{\rho }\hat{\nu }\right)}_{f}c},\;\hat{N}=\frac{{\hat{N}}^{*}}{c},\;\hat{T}=\frac{{\hat{T}}^{*}-{\hat{T}}_{\infty }}{{\hat{T}}_{w}-{\hat{T}}_{\infty }},\;$$

where ${\hat{\nu }}_{f}$ describes the kinematic viscosity of water. Using above transformations, dimensionless forms of Equations (1)–(5), (9) and (10) are:

(12) $$\frac{\partial \hat{u}}{\partial \hat{x}}+\frac{\partial \hat{v}}{\partial \hat{y}}=0,$$

(13) $$\hat{u}\frac{\partial \hat{u}}{\partial \hat{x}}+\hat{v}\frac{\partial \hat{u}}{\partial \hat{y}}+\frac{{\hat{\rho }}_{f}}{{\hat{\rho }}_{nf}}\frac{\partial \hat{p}}{\partial \hat{x}}=\frac{{\hat{\rho }}_{f}}{{\hat{\rho }}_{nf}}\left(\frac{1}{{\left(1-\phi \right)}^{2.5}}+K\right)\frac{\partial^{2}\hat{u}}{\partial {\hat{y}}^{2}}+\frac{{\hat{\rho }}_{f}}{{\hat{\rho }}_{nf}}K\frac{\partial \hat{N}}{\partial \hat{y}},$$

(14) $$\hat{u}\frac{\partial \hat{v}}{\partial \hat{x}}+\hat{v}\frac{\partial \hat{v}}{\partial \hat{y}}+\frac{{\hat{\rho }}_{f}}{{\hat{\rho }}_{nf}}\frac{\partial \hat{p}}{\partial \hat{y}}=\frac{{\hat{\rho }}_{f}}{{\hat{\rho }}_{nf}}\left(\frac{1}{{\left(1-\phi \right)}^{2.5}}+K\right)\frac{\partial^{2}\hat{v}}{\partial {\hat{y}}^{2}}-\frac{{\hat{\rho }}_{f}}{{\hat{\rho }}_{nf}}K\frac{\partial \hat{N}}{\partial \hat{x}}$$

(15) $$\hat{u}\frac{\partial \hat{N}}{\partial \hat{x}}+\hat{v}\frac{\partial \hat{N}}{\partial \hat{y}}=-K\frac{{\hat{\rho }}_{f}}{{\hat{\rho }}_{nf}}\left(2\hat{N}+\frac{\partial \hat{u}}{\partial \hat{y}}\right)+\frac{{\hat{\rho }}_{f}}{{\hat{\rho }}_{nf}}\left(\frac{1}{{\left(1-\phi \right)}^{2.5}}+\frac{K}{2}\right)\frac{\partial^{2}\hat{N}}{\partial {\hat{y}}^{2}}$$

(16) $$\hat{u}\frac{\partial \hat{T}}{\partial \hat{x}}+\hat{v}\frac{\partial \hat{T}}{\partial \hat{y}}=\frac{{\hat{\alpha }}_{nf}}{{\hat{\nu }}_{f}}\frac{\partial^{2}\hat{T}}{\partial {\hat{y}}^{2}}$$

(17) $$\hat{u}=\hat{x},\hat{v}=0,\hat{N}=-n\frac{\partial \hat{u}}{\partial \hat{y}},\hat{T}=1\;at\;\hat{y}=0$$

(18) $$\hat{u}=(\frac{a}{c})\hat{x}+(\frac{b}{c})\hat{y},\hat{N}=-(\frac{b}{c}),\hat{T}=0\;when\;\hat{y}\to \infty$$

where $K=\frac{\hat{k}}{{\hat{\mu }}_{f}}$ denote the micropolar strengthening parameter and $B=\frac{a}{c}$ is the stretching ratio parameter. Relation between stream function and velocity components is given below as [22]:

(19) $$\hat{u}=\frac{\partial \psi }{\partial \hat{y}},\hat{v}=-\frac{\partial \psi }{\partial \hat{x}}$$

By means of stream function and fact ${\hat{p}}_{\hat{x}\hat{y}}={\hat{p}}_{\hat{y}\hat{x}}$ for elimination of $\hat{p}$, Equations (12)–(18) become:

(20) $$\frac{{\hat{\rho }}_{f}}{{\hat{\rho }}_{nf}}\left({\left(1-\phi \right)}^{-2.5}+K\right)\frac{\partial^{2}}{\partial {\hat{y}}^{2}}\left(\nabla^{2}\psi \right)+\frac{{\hat{\rho }}_{f}}{{\hat{\rho }}_{nf}}K\nabla^{2}\hat{N}+\frac{\partial \left(\psi ,\nabla^{2}\psi \right)}{\partial \left(\hat{x},\hat{y}\right)}=0,$$

(21) $$\frac{\partial \psi }{\partial \hat{y}}\frac{\partial \hat{N}}{\partial \hat{x}}-\frac{\partial \psi }{\partial \hat{x}}\frac{\partial \hat{N}}{\partial \hat{y}}=-K\frac{{\hat{\rho }}_{f}}{{\hat{\rho }}_{nf}}\left(2\hat{N}+\frac{\partial^{2}\psi }{\partial {\hat{y}}^{2}}\right)+\frac{{\hat{\rho }}_{f}}{{\hat{\rho }}_{nf}}\left({\left(1-\phi \right)}^{-2.5}+\frac{K}{2}\right)\frac{\partial^{2}\hat{N}}{\partial {\hat{y}}^{2}}$$

(22) $$Pr\left(\frac{\partial \psi }{\partial \hat{y}}\frac{\partial \hat{T}}{\partial \hat{x}}-\frac{\partial \psi }{\partial \hat{x},}\frac{\partial \hat{T}}{\partial \hat{y}}\right)=\frac{{\hat{\alpha }}_{nf}}{{\hat{\alpha }}_{f}}\frac{\partial^{2}\hat{T}}{\partial {\hat{y}}^{2}}$$

where $Pr=\frac{{\hat{\nu }}_{f}}{{\hat{\alpha }}_{f}}$ embodies the Prandtl number. Stream function transforms the boundary conditions as:

(23) $$\psi =0,\;\;\frac{\partial \psi }{\partial \hat{y}}=\hat{x},\hat{N}=-n\frac{\partial^{2}\psi }{\partial {\hat{y}}^{2}},\hat{T}=1\;\;\;\;\mathrm{at}\;\hat{y}=0,$$

(24) $$\psi =0.5\gamma {\hat{y}}^{2}+B\hat{x}\hat{y},\hat{N}=-\left(\frac{b}{c}\right),\;\;\;\;\hat{T}=0\;\;\mathrm{as}\;\hat{y}\to \infty .$$

where flow obliqueness is identified by parameter $\gamma =\frac{b}{c}$. ψ [22] is redefined as:

(25) $$\psi \left(\hat{x},\hat{y}\right)=G\left(\hat{y}\right)+\hat{x}F\left(\hat{y}\right),\;\;\;\hat{N}\left(\hat{x},\hat{y}\right)=S\left(\hat{y}\right)+\hat{x}J\left(\hat{y}\right),\hat{T}\left(\hat{x},\hat{y}\right)=\theta \left(\hat{y}\right),$$

where normal, tangential components of flow and micro-rotation are denoted by $F\left(\hat{y}\right)$, $G\left(\hat{y}\right),J\left(\hat{y}\right)$ and $S\left(\hat{y}\right)$, respectively. Employing Equation (25) in Equations (20)–(24) and integrating once we have:

(26) $$\frac{{\hat{\rho }}_{f}}{{\hat{\rho }}_{nf}}\left(KJ^{\prime }+\left({\left(1-\phi \right)}^{-2.5}+K\right)F^{\prime \prime \prime }\right)+FF^{\prime \prime }-{\left(F^{\prime }\right)}^{2}+C_{1}=0,$$

(27) $$\frac{{\hat{\rho }}_{f}}{{\hat{\rho }}_{nf}}\left(KS^{\prime }+\left({\left(1-\phi \right)}^{-2.5}+K\right)G^{\prime \prime \prime }\right)+{FG}^{\prime \prime }-F^{\prime }G^{\prime }+C_{2}=0,$$

(28) $$\left({\left(1-\phi \right)}^{-2.5}+0.5K\right)J^{\prime \prime }-K\left(2J+F^{\prime \prime }\right)+\frac{{\hat{\rho }}_{nf}}{{\hat{\rho }}_{f}}\left({FJ}^{\prime }-F^{\prime }J\right)=0$$

(29) $$\left({\left(1-\phi \right)}^{-2.5}+0.5K\right)S^{\prime \prime }-K\left(2S+G^{\prime \prime }\right)+\frac{{\hat{\rho }}_{nf}}{{\hat{\rho }}_{f}}\left({FS}^{\prime }-G^{\prime }J\right)=0,$$

(30) $$\frac{{\hat{k}}_{nf}}{{\hat{k}}_{f}}\theta^{\prime \prime }+Pr.F\theta^{\prime }\left(1-\phi +\frac{{\left(\hat{\rho }c_{\hat{p}}\right)}_{s}}{{\left(\hat{\rho }c_{\hat{p}}\right)}_{f}}\phi \right)=0.$$

Here, constants $C_{1}$ and $C_{2}$ are introduced due to integration and primes are representing rates of change w.r.t $\hat{y}$. Conditions at boundaries take the form:

(31) $$\left.\begin{array}{c} F\left(0\right)=0,F^{\prime }\left(0\right)=1,-{nF}^{\prime \prime }\left(0\right)=J\left(0\right),-nG^{\prime \prime }\left(0\right)=S\left(0\right),\theta \left(0\right)=1,\\ F^{\prime }\left(\infty \right)=\frac{a}{c},\;\;G^{\prime \prime }\left(\infty \right)=\gamma ,J\left(\infty \right)=0,S\left(\infty \right)=-\frac{b}{c},\theta \left(\infty \right)=0. \end{array}\right\}$$

Equation (26) gives $C_{1}={\left(\frac{a}{c}\right)}^{2}$ by implementing the limit $\hat{y}\to \infty$ and using $F^{\prime }\left(\infty \right)=\frac{a}{c}$. An analysis of Equation (26) in terms of boundary layer theory specifies that $F\left(\hat{y}\right)=\left(\frac{a}{c}\right)\hat{y}+A$ when $\hat{y}$ approaches infinity, where constant A signifies displacement of boundary layer. $C_{2}=-A\gamma$ is attained by means of the limit when $\hat{y}$ approaches infinity and condition $G^{\prime \prime }\left(\infty \right)=\gamma$ used in Equation (27). Hence Equations (26) and (27) are transformed as:

(32) $$F^{\prime \prime \prime }\left({\left(1-\phi \right)}^{-2.5}+K\right)+KJ^{\prime }+\frac{{\hat{\rho }}_{nf}}{{\hat{\rho }}_{f}}\left({FF}^{\prime \prime }-{\left(F^{\prime }\right)}^{2}+{\left(\frac{a}{c}\right)}^{2}\right)=0,$$

(33) $$G^{\prime \prime \prime }\left({\left(1-\phi \right)}^{-2.5}+K\right)+KS^{\prime }+\frac{{\hat{\rho }}_{nf}}{{\hat{\rho }}_{f}}\left(FG^{\prime \prime }-F^{\prime }G^{\prime }-A\gamma \right)=0,$$

Let us introduce another relation:

(34) $$\frac{G^{\prime }\left(\hat{y}\right)}{H\left(\hat{y}\right)}=\gamma$$

Using Equation (34) in Equations (27) and (33):

(35) $$H^{\prime \prime }\left({\left(1-\phi \right)}^{-2.5}+K\right)+\frac{K}{\gamma }S^{\prime }+\frac{{\hat{\rho }}_{nf}}{{\hat{\rho }}_{f}}\left({FH}^{\prime }-F^{\prime }H-A\right)=0,$$

(36) $$S^{\prime \prime }\left({\left(1-\phi \right)}^{-2.5}+\frac{K}{2}\right)-K\left(2S+\gamma H^{\prime }\right)+\frac{{\hat{\rho }}_{nf}}{{\hat{\rho }}_{f}}\left({FS}^{\prime }-\gamma HJ\right)=0,$$

(37) $$H\left(0\right)=0,H^{\prime }\left(\infty \right)=1.$$

### Physical Measures of Concern

${{\hat{\tau }}^{*}}_{\omega }={\left[\left({\hat{\mu }}_{nf}+\hat{k}\right)\frac{\partial {\hat{u}}^{*}}{\partial {\hat{y}}^{*}}+\hat{k}{\hat{N}}^{*}\right]}_{{\hat{y}}^{*}=0}$ and ${{\hat{q}}^{*}}_{\omega }={-\hat{k}}_{nf}{\left(\frac{\partial {\hat{T}}^{*}}{\partial {\hat{y}}^{*}}\right)}_{{\hat{y}}^{*}=0}$ are the dimensional forms of surface shear stress and heat transfer rate. Their dimensionless forms are:

(38) $${\hat{\tau }}_{\omega }=\left(xF^{\prime \prime }\left(0\right)+\gamma H^{\prime }\left(0\right)\right)\left({\left(1-\phi \right)}^{-2.5}+K\left(1-n\right)\right),$$

(39) $${\hat{q}}_{\omega }=-\theta^{\prime }\left(0\right)\left(\frac{{\hat{k}}_{nf}}{{\hat{k}}_{f}}\right).$$

## 3. Numerical Solution

Using the following substitutions in the set of coupled non-linear ODE’s (28), (30), (32), (35)–(36) along with conditions (31) and (37):

(40) $$\left.\begin{array}{c} F=w_{1},F^{\prime }={w^{\prime }}_{1}=w_{2},F^{\prime \prime }={w^{\prime }}_{2}=w_{3}\\ H=w_{4},H^{\prime }={w^{\prime }}_{4}=w_{5,}J^{\prime }={w^{\prime }}_{6}=w_{7},S=w_{8}\\ S^{\prime }={w^{\prime }}_{8}=w_{9},\theta =w_{10},\theta^{\prime }={w^{\prime }}_{10}=w_{11} \end{array}\right\}$$

We have:

(41) $${w^{\prime }}_{3}=\frac{-1}{a\left(\phi \right)+K}\left(Kw_{7}+\frac{{\hat{\rho }}_{nf}}{{\hat{\rho }}_{f}}\left(w_{1}w_{3}-{w_{2}}^{2}+{\left(\frac{a}{c}\right)}^{2}\right)\right),$$

(42) $${w^{\prime }}_{5}=\frac{-1}{a\left(\phi \right)+K}\left(\frac{K}{\gamma }w_{9}+\frac{{\hat{\rho }}_{nf}}{{\hat{\rho }}_{f}}\left(w_{1}w_{5}-w_{2}w_{4}-A\right)\right)$$

where $a\left(\phi \right)={\left(1-\phi \right)}^{-2.5}$:

(43) $${w^{\prime }}_{7}=\frac{1}{a\left(\phi \right)+\frac{K}{2}}\left(K\left(2w_{6}+w_{3}\right)-\frac{{\hat{\rho }}_{nf}}{{\hat{\rho }}_{f}}\left(w_{1}w_{7}-w_{2}w_{6}\right)\right),$$

(44) $${w^{\prime }}_{9}=\frac{1}{a\left(\phi \right)+\frac{K}{2}}\left(K\left(2w_{8}+{\gamma w}_{5}\right)-\frac{{\hat{\rho }}_{nf}}{{\hat{\rho }}_{f}}\left(w_{1}w_{9}-{\gamma w}_{4}w_{6}\right)\right)$$

(45) $${w^{\prime }}_{11}=-Pr\frac{{\hat{k}}_{f}}{{\hat{k}}_{nf}}\frac{{\left(\hat{\rho }c_{\hat{p}}\right)}_{nf}}{{\left(\hat{\rho }c_{\hat{p}}\right)}_{f}}\left(w_{1}w\right),$$

(46) $$\left.\begin{array}{c} w_{1}\left(0\right)=0,w_{2}\left(0\right)=1,w_{3}\left(0\right)=-\frac{b_{1}}{n},w_{4}\left(0\right)=0,w_{5}\left(0\right)=-\frac{b_{2}}{n\gamma },w_{6}\left(0\right)=b_{1}\\ w_{7}\left(0\right)=b_{3},w_{8}\left(0\right)=b_{2},\;\;w_{9}\left(0\right)=b_{4},\;\;w_{10}\left(0\right)=1,w_{11}\left(0\right)=b_{5} \end{array}\right\}.$$

IVP (initial value problem) obtained by above substitution is numerically solved by Range–Kutta–Fehlberg fourth order scheme combined with shooting technique [14]. Where involved shooting parameters $b_{1},b_{2},b_{3},b_{4}\;\mathrm{and}\;b_{5}$ are assessed with Newton Raphson’s method. A convergence standard chosen for numerical outcomes is $10^{-6}$.

## 4. Discussion and Results

**Figure 2:**
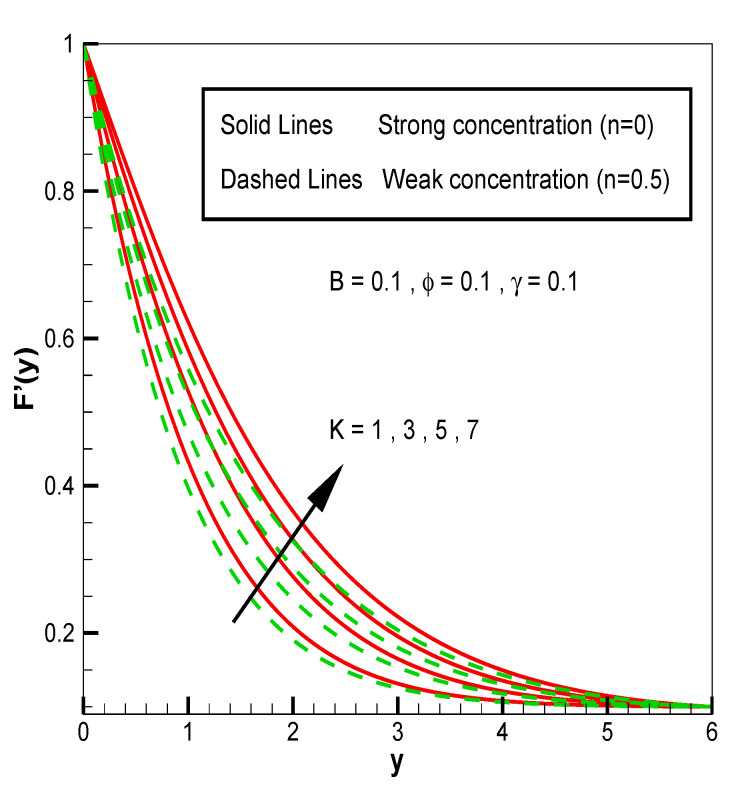
$F^{\prime }\left(y\right)$ for material parameter K.

**Figure 3:**
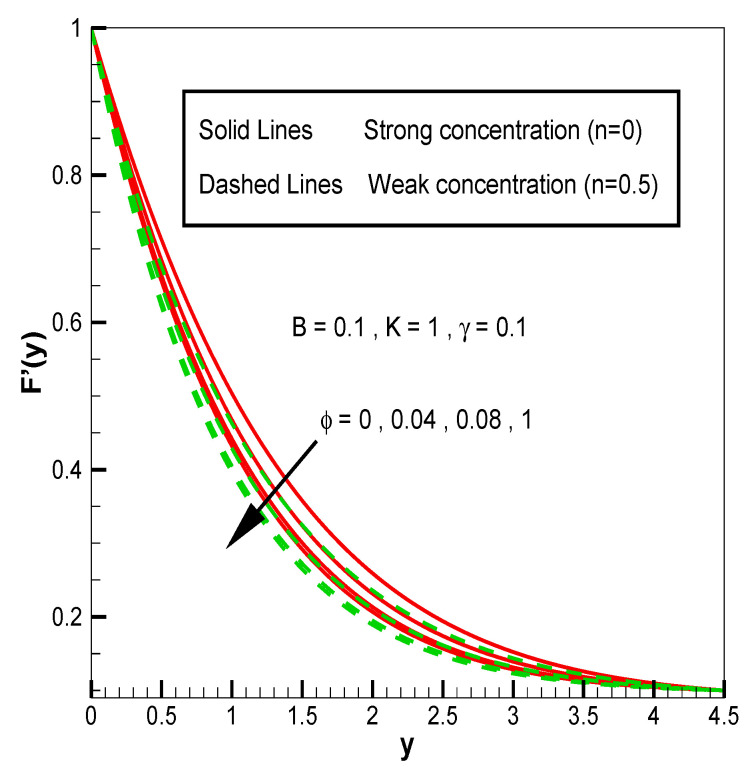
$F^{\prime }\left(y\right)$ for volume fraction of nanoparticles ϕ.

**Figure 4:**
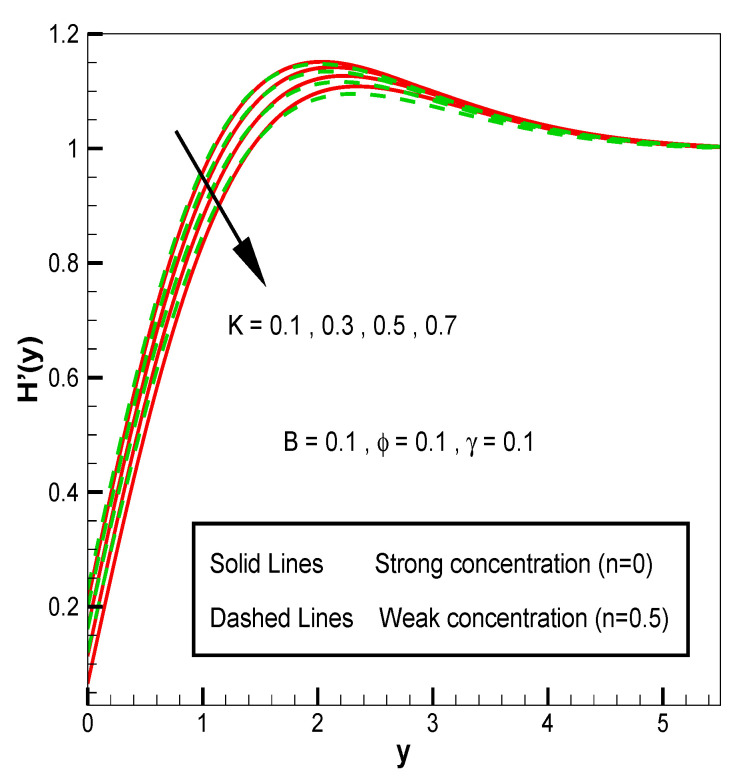
$H^{\prime }\left(y\right)$ for material parameter **K**.

**Figure 5:**
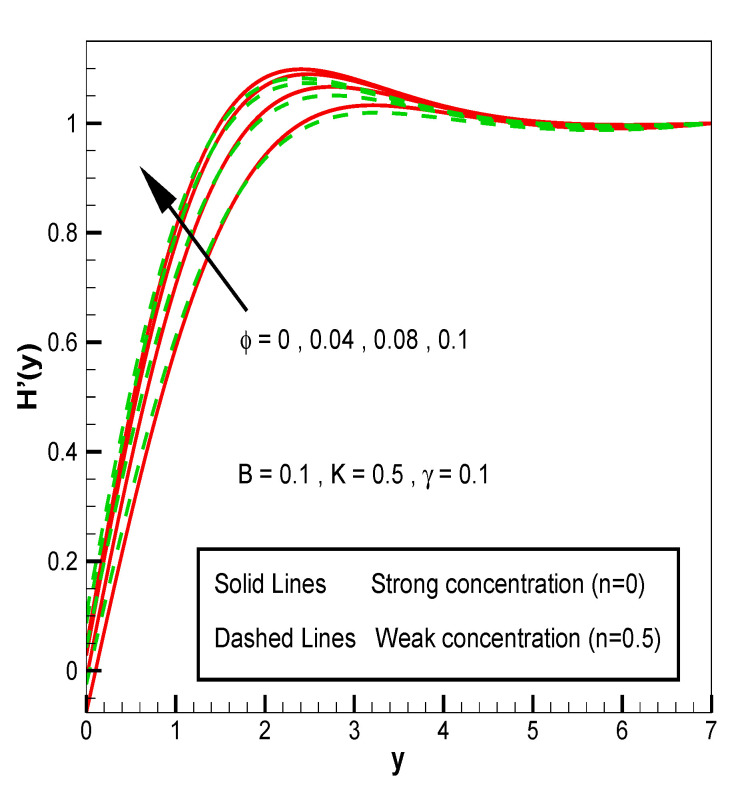
$H^{\prime }\left(y\right)$ for volume fraction of nanoparticles ϕ.

**Figure 6:**
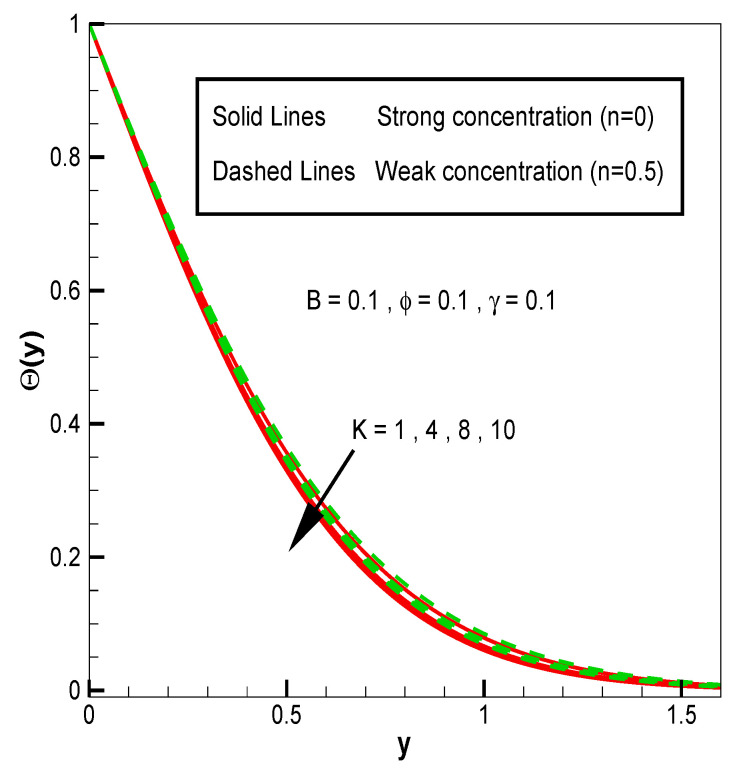
$\theta \left(y\right)$ for material parameter K.

**Figure 7:**
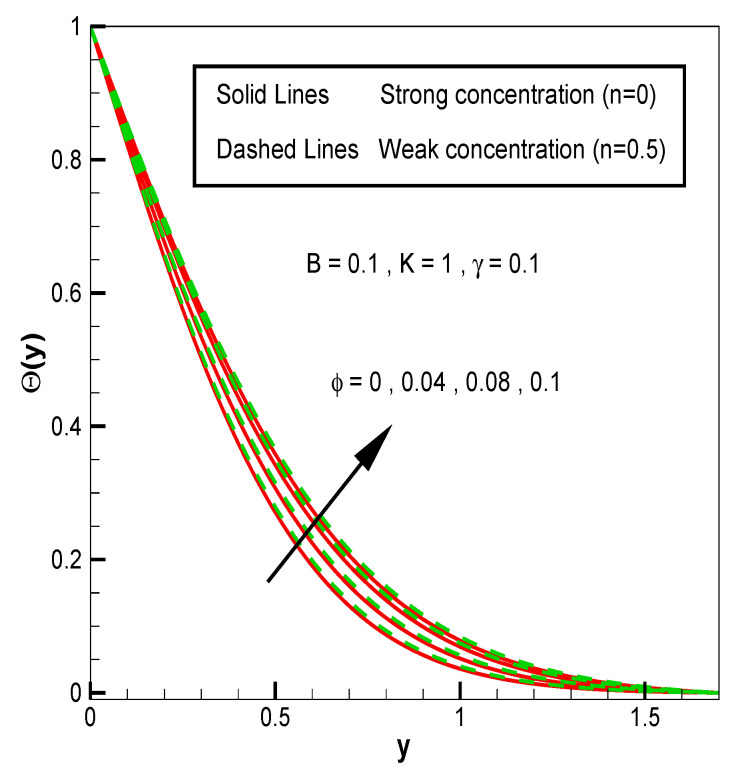
$\theta \left(y\right)$ for volume fraction of nanoparticles ϕ.

**Figure 8:**
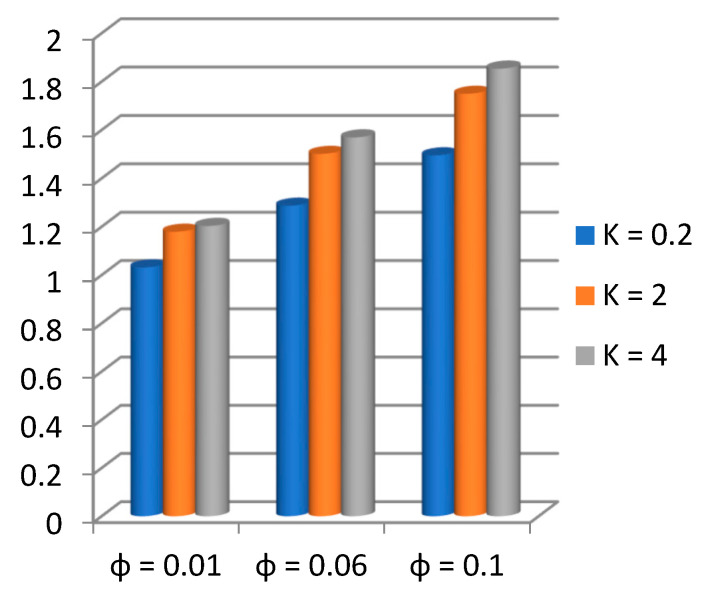
Magnitude of shear stress $\left(\frac{1}{{\left(1-\phi \right)}^{2.5}}+K\left(1-n\right)\right)\left(F^{\prime \prime }\left(0\right)+\gamma H^{\prime }\left(0\right)\right)$ at the surface with x=1 against ϕ.

**Figure 9:**
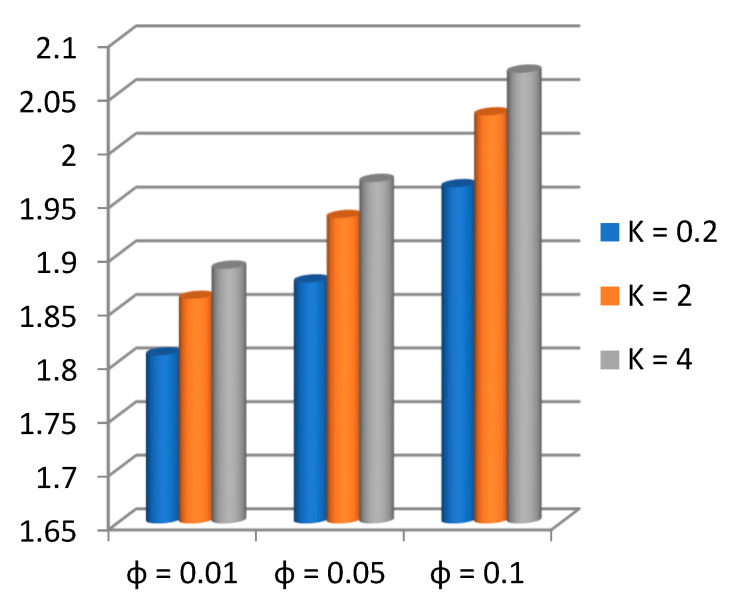
Local heat flux ${\hat{q}}_{\omega }=-\left(\frac{{\hat{k}}_{nf}}{{\hat{k}}_{f}}\right)\theta^{\prime }\left(0\right)$ at the surface against ϕ.

**Figure 10:**
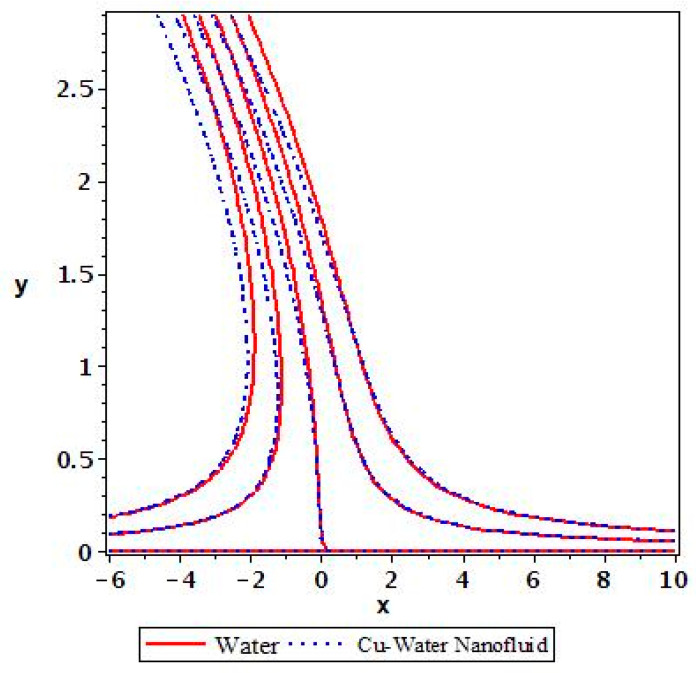
Streamlines for ϕ=0 and ϕ=0.1 with γ=1.

**Figure 11:**
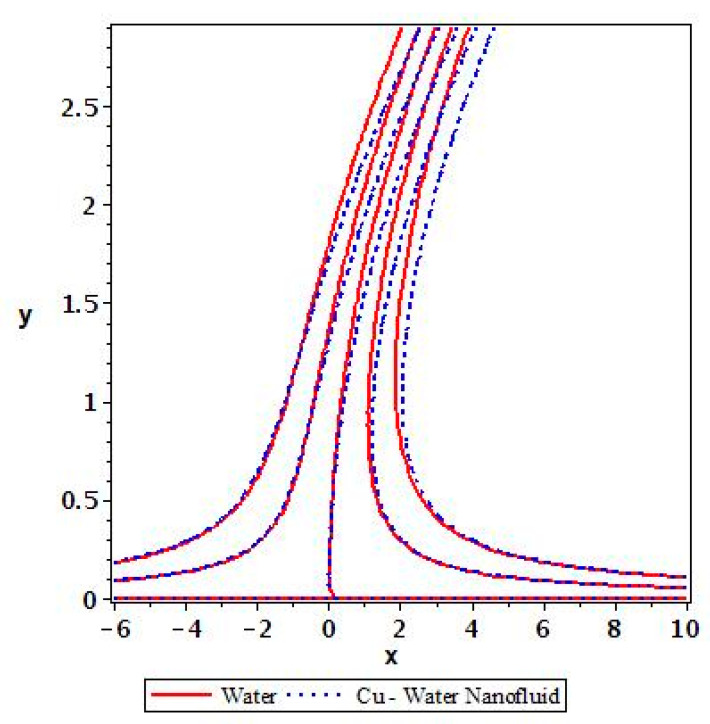
Streamlines for ϕ=0 and ϕ=0.1 with γ=−1.

Applying the above-mentioned numerical scheme, computation with various values of micropolar coupling constant K, nanoparticles volume fraction ϕ has been carried out for two cases, (n=0) when concentration is strong and (n=0.5) when concentration of microelements is weak. When n=0, there is no rotation for microelements that are close to walls. This situation corresponds to concentrated particle flows. We also have considered other particular cases, such as n=0.5, which represent the weak concentration and the turbulent flow, respectively. Nanoliquid-containing copper nanoparticles and water as carrier liquid has been taken in the current flow problem. Thermophysical properties of Cu and water are given in Table 1. Variation in $F^{\prime }\left(y\right)$ (normal velocity profile) of micropolar nanofluid flow is plotted in Figure 2 by varying the values of micropolar coupling parameter K. In this plot, we perceived that $F^{\prime }\left(y\right)$ increases with material parameter K. This is for the reason that material parameter is the ratio of vortex viscosity and dynamic viscosity of nanofluid. By increasing K we mean dynamic viscosity of nanofluid is decreasing more than the vortex viscosity and fluid is facing less resistive forces; that is why its normal velocity profile enhances. Furthermore, it is found that normal velocity profile for (n=0) strong concentration is higher in magnitude than $\left(n=0.5\right)$ weak concentration. When the value of n is increased, i.e., the concentration of the micro-element is reduced, the velocity profile is enhanced. The situation when concentration is strong, is that the density of microelements is sufficiently large and they are unable to rotate, due to which their normal velocity profile is higher in magnitude. It can also be witnessed that there is no significant variation in the momentum boundary layer thickness in this figure. Figure 3 depicts the normal profile of velocity $F^{\prime }\left(y\right)$ with variation in volume fraction ϕ of copper nanoparticles. It is noticed that ϕ has declined the normal profile of velocity $F^{\prime }\left(y\right).$ It may be due to the resistive forces that emerge for the higher values of ϕ in the flow, which drops the normal velocity flow profile. It is also noticed that $F^{\prime }\left(y\right)$ for strong concentration is higher when compared with this profile for weak concentration. The analysis of $H^{\prime }\left(y\right)$ (tangential velocity profile) for various values of emerging parameters have been performed in Figure 4 and Figure 5. Figure 4 depicts that micropolar coupling parameter K and concentration effects are quite contrasting on tangential profile when compared with normal profile. K has decreasing effect on it, while this velocity profile with weak concentration is higher in magnitude than strong concentration, but this behaviour is reversed away from the surface. In the same way as K, quite the opposite effects of ϕ are seen on the tangential component of velocity in Figure 5 when matched with normal velocity profile. It is perceived that volume fraction ϕ of copper nanoparticles has an increasing effect on tangential velocity profile $H^{\prime }\left(y\right).$ It is also detected that near the wall, $H^{\prime }\left(y\right)$ with weak concentration is marginally advanced when compared with the case for strong concentration, but far from the wall, the opposite behaviour is witnessed. Variations in temperature distribution $\theta \left(y\right)$ influenced by pertinent parameters are displayed in Figure 6 and Figure 7. The temperature decreases with material parameter K, while it enhances with nanoparticles volume fraction ϕ. By increasing ϕ, we are increasing the thermal conductivity of nanofluids, which cause a rise in temperature profile. In both cases, temperature profile with strong concentration is lower than weak concentration. Figure 8 is plotted to investigate the behavior of shear stress magnitude $\left(\frac{1}{{\left(1-\phi \right)}^{2.5}}+K\left(1-n\right)\right)\left(xF^{\prime \prime }\left(0\right)+\gamma H^{\prime }\left(0\right)\right)$ at the extending surface when it is plotted against ϕ for different values of material parameter K with x=1. This bar graph exhibits that rise in material parameter K, which increases the stress at the surface; likewise, ϕ has increasing impact on it. Similarly, in Figure 9 we note that material parameter enhances the local heat flux ${\hat{q}}_{\omega }=-\left(\frac{{\hat{k}}_{nf}}{{\hat{k}}_{f}}\right)\theta^{\prime }\left(0\right)$ and nanoparticles volume fraction ϕ also increasingly influences the heat transfer rate. This shows that heat transfer rate grows with ϕ and K. The apparent reason behind this kind of temperature response is that increase is volume fraction of tiny nano particles consequently raises the temperature of the fluid, which, in turn, upsurges the corresponding surface temperature gradient. The same is found in the case observed for surface skin friction. The obvious reason is that enhancing volume fraction leads to more friction within the fluid, which results in higher surface skin friction coefficient. Noticeable effects of nanoparticles volume fraction ϕ on streamlines of the flow are presented through Figure 10 and Figure 11. It is found that streamlines of nanofluid-containing cu nanoparticles volume fraction (ϕ=0.1) are more tilted towards the left, as compared with streamlines of pure fluid (water) in the absence of cu nanoparticles (ϕ=0) for the case of obliqueness angle γ=1. Moreover, it is noticed that for the case of obliqueness angle γ=−1, streamlines with cu nanoparticles are more slanted to the right side.

A comparison of present and previously published results by Mahapatra [35] and Pop [36] is shown in Table 2. An admirable agreement is noted between both outcomes. Some numerical outcomes of shear stress and heat flux for various values of nanoparticles volume fraction ϕ and material constant K are shown in Table 3, whereas other parameters are kept fixed. It is very obvious that shear stress declined, whereas heat transfer rate sufficiently enhanced with increasing nanoparticles volume fraction. Micropolar coupling parameter also had a decreasing effect on shear stress, but it has not prominently influenced the heat transfer rate for higher values $\left(K=2,3,4\right),$ as compared to smaller values $\left(K=0.1,1.5\right).$

## 5. Conclusions

The present investigation focuses on the effects of microelements concentration n, micropolar coupling or material constant K and volume fraction ϕ of copper nanoparticles on micropolar nanofluid. Core outcomes from the obtained results are presented below:

- Material constant K and nanoparticles volume fraction ϕ depicts the opposite influence on tangential and normal profiles of velocity.
- Higher temperature profiles are witnessed when low concentration is considered.
- Magnitude of shear stress and heat transfer rate at elongating surface elevated with ϕ.
- Material parameter K has increasingly influenced the shear stress and rate of heat transfer.
- Normal velocity profile is higher in magnitude for the case of strong concentration (n=0) of microelements, whereas tangential velocity profile is higher near the surface for the case of weak concentration (n=0.5) of microelements.
- An increase of 3.74% in heat flux is observed when the volume fraction of nanoparticles is increased from 1 to 5%.

## Figures and Tables

**Table 1:** Thermal and physical characteristics of copper and water.

Thermo-Physical Properties	$c_{\hat{p}}\left(\frac{J}{kg.K}\right)$	$\hat{\rho }\left(\frac{kg}{m^{3}}\right)$	$\hat{k}\left(\frac{W}{m.K}\right)$
$H_{2}O$	4179	997.1	0.613
Cu	385	8933	400

**Table 2:** Comparison table for stretching ratio B=a/c when ϕ=n=K=0.

$F^{\prime \prime }(0)$				$H^{\prime }(0)$		$-\theta^{\prime }(0)$	
a/c	Present	Mahapatra **[35]**	Pop **et al. [36]**	Present	Pop **et al. [36]**	Present	Pop **et al. [36]**
0.1	−0.96938	−0.9694	−0.96938	0.26341	0.26278	0.60215	0.60281
0.3	−0.84937		−0.84942	0.60631	0.60573	0.64728	0.64732
0.8	−0.29937		−0.29938	0.93472	0.93430	0.75709	0.75709
1.0	0.0		0.0	1.0	1.0	0.79788	0.79788
2.0	2.01750	2.01750	2.01750	1.16522	1.16489	0.97872	0.97872
3.0	4.72928	4.72930	4.72928	1.23465	1.23438	1.13209	1.13209

**Table 3:** Shear stress and heat flux for nanoparticles volume fraction Φ and material parameter **K** when (**B** = 0.1, **n** − 0.5, **K** = 0.1, γ=0.1).

Φ	${\hat{\tau }}_{\omega }$	${\hat{q}}_{\omega }$	K	${\hat{\tau }}_{\omega }$	${\hat{q}}_{\omega }$
0.01	−1.01321	1.80220	0.1	−1.47192	1.95798
0.05	−1.21460	1.86971	1.5	−1.70149	2.01619
0.1	−1.47192	1.95798	2	−1.74958	2.03035

## Data Availability

Not applicable.

## Nomenclature

$\hat{u}$	$\hat{x}$-component of velocity $\left(m/s\right)$
$\hat{v}$	$\hat{y}$-component of velocity $\left(m/s\right)$
$\hat{T}$	Temperature $\left(K\right)$
${\hat{T}}_{w}$	Wall temperature $\left(K\right)$
${\hat{T}}_{\infty }$	Ambient temperature $\left(K\right)$
$\hat{p}$	Pressure $\left(N/m^{2}\right)$
$\hat{x},\hat{y}$	Cartesian coordinates along the stretching surface and normal to it $\left(m\right)$
$c_{\hat{p}}$	Specific heat $\left(\frac{J}{Kg.K}\right)$
$\hat{k}$	Vortex viscosity
K	Micropolar strengthening parameter
j	Micro inertia density $\left(Kg/m^{3}\right)$
B=a/c	Stretching ratio parameter
n	Micro element concentration
$\hat{N}$	Micro-rotation vector
m	Variable viscosity parameter
Pr	Prandtl number
**Greek Symbols**	
ϕ	Solid volume fraction of nanoparticles
$\mu_{0}$	Reference viscosity $\left(\frac{Kg}{m.s}\right)$
$\hat{\nu }$	Kinematics viscosity $\left(m^{2}/s\right)$
$\hat{\rho }$	Density $\left(Kg/m^{3}\right)$
γ=b/c	Flow obliqueness parameter
$\hat{\mu }$	Dynamic viscosity $\left(\frac{Kg}{m.s}\right)$
**Subscripts**	
f	Base fluid
s	Nanoparticles
nf	Nanofluid
